# Defect Classification for Additive Manufacturing with Machine Learning

**DOI:** 10.3390/ma16186242

**Published:** 2023-09-16

**Authors:** Mika León Altmann, Thiemo Benthien, Nils Ellendt, Anastasiya Toenjes

**Affiliations:** 1 Leibniz Institute for Materials Engineering—IWT, Badgasteiner Straße 3, 28359 Bremen, Germany; altmann@iwt-bremen.de (M.L.A.); thiemo@uni-bremen.de (T.B.); ellendt@iwt.uni-bremen.de (N.E.); 2 Faculty of Production Engineering, University of Bremen, Badgasteiner Straße 1, 28359 Bremen, Germany

**Keywords:** PBF-LB/M, Ti6Al4V, defect classification, machine learning, additive manufacturing

## Abstract

Additive manufacturing offers significant design freedom and the ability to selectively influence material properties. However, conventional processes like laser powder bed fusion for metals may result in internal defects, such as pores, which profoundly affect the mechanical characteristics of the components. The extent of this influence varies depending on the specific defect type, its size, and morphology. Furthermore, a single component may exhibit various defect types due to the manufacturing process. To investigate these occurrences with regard to other target variables, this study presents a random forest tree model capable of classifying defects in binary images derived from micrographs. Our approach demonstrates a classification accuracy of approximately 95% when distinguishing between keyhole and lack of fusion defects, as well as process pores. In contrast, unsupervised models yielded prediction accuracies below 60%. The model’s accuracy in differentiating between lack of fusion and keyhole defects varies based on the manufacturing process’s parameters, primarily due to the irregular shapes of keyhole defects. We provide the model alongside this paper, which can be utilized on a standard computer without the need for in situ monitoring systems during the additive manufacturing process.

## 1. Introduction

The laser powder bed fusion of metals is an additive manufacturing process in which components with complex geometries are produced layer by layer [1,2]. In this process, a thin layer of powder is applied with a thin blade and locally melted by a laser. Both steps are repeated until the final height of the parts is reached [3,4].

The mechanical properties of additive manufactured components, as in powder metallurgy components, depend significantly on the relative density and the contained defect shapes as well as their relative proportions [5,6]. Various defect types form due to distinct process variables, leading to a substantial impact on relative density. Specifically, laser power, scanning speed, and scanning strategy play pivotal roles in this regard as well as the size and morphology distribution of the powder [7,8]. Many defects and microstructural features show a correlation with the applied energy density [9,10]. This energy density can be described with major process parameters as

(1) $$ED=\frac{P_{L}}{v_{S}\cdot d_{H}\cdot D_{L}}.$$

Here, $P_{L}$ is the laser power in W, $v_{S}$ is the scan speed in mm/s, $d_{H}$ is the hatch distance in mm, and $D_{L}$ is the layer thickness in mm [11]. Beyond the stable process window, defects like inadequate track or layer bonding, referred to as “lack of fusion”, may occur [12]. Defects can be classified based on their morphologies and formation mechanisms. For example, gas or process pores are usually small (mostly smaller than 100 µm), approximately spherical voids in the material. They can result from different causes. For instance, a lower powder packing density leads to larger gas accumulations between the particles. These trapped gases remain enclosed within the melt and cannot escape the melt pool due to rapid cooling rates. Additional causes may include hollow powder particles or the vaporization of metal at elevated local temperatures. These porosities exhibit random distributions within the component and pose challenges in terms of prevention [6].

Lacks of fusion defects resulted from incomplete fusions of the powder particles. The main cause is insufficient energy input. Thus, the proportions of lacks of fusion defects increase with the exposure speed and decreases with increasing laser power [13]. Two subgroups can be distinguished: Bonding defects due to insufficiently melted materials and inclusions of unmelted powder. Insufficient energy input results in a small-sized melt pool and, consequently, a narrower melt track width. This results in a lack of trace overlap. Accordingly, these defects usually occur between melt tracks or the layers [6]. Excessive energy input can lead to the temperature within the melt track surpassing the material’s boiling point, causing portions of the material to vaporize [14]. The vapor pressure pushes the melt to the edge of the melt pool, forming narrow vapor capillaries [14,15]. Within this keyhole mode, relatively large gas pores may occur [16,17]. The high energy further leads to a longer lifetime of the melt pool, allowing smaller gas pores to combine into larger ones [17,18]. High-energy input and rapid cooling rates induce significant temperature gradients and thermal stresses within additive manufactured components. These conditions can lead to the formation of defects that serve as initial points for cracks. Subsequently, these cracks propagate throughout the component under the influence of thermal stresses [6].

Defects have a substantial impact on the mechanical properties of additively manufactured components, causing reduced elongation at fracture in comparison to components made through conventional methods like casting or machining. When under mechanical loads, these defects create stress concentrations, leading to the initiation of cracks. The morphologies, numbers, sizes, and positions of the pores, in particular, influence the properties of the components. For example, spherical defects have less influence than non-uniformly shaped defects [6]. Already 1 vol.% of defects reduce the mechanical properties enormously, e.g., the elongation until break by ~25%. At defects of up to 5 vol.%, the properties are already so poor that application under mechanical load is usually not possible, e.g., the elongation until break is reduced by ~60%, and the fatigue limit is reduced by ~70% [6]. By simulating the influence of defects such as keyhole and lack of fusion defects on fatigue properties, it was shown that gas pores and keyhole defects converge with the results of defect free parts at high stress levels. On low stress levels, parts containing lacks of fusion defects achieve similar or higher fatigue lives than defect free or those with gas pores/keyhole defects [19].

As previously discussed, internal defects hold significant importance in the application of additively manufactured components, as they have a substantial impact on their mechanical properties. Specifically, lacks of fusion defects exacerbate mechanical properties due to their irregular and, at times, sharp-edged contours. In contrast, keyhole defects have a lesser influence on mechanical properties, primarily owing to their more convex contours [6]. Hence, it is necessary to obtain a better understanding about process windows and their influences on defects, as well as their influence on component properties [20].

The first step involves defect classification. To automate this process and extract more comprehensive insights about these defects, methods from image analysis, statistics, and data science can be applied. Data science includes methods to extract hidden patterns and features from large data sets. These findings can be subsequently employed to make predictions for future occurrences, underscoring the necessity for efficient and effective methods and algorithms [21,22]. Machine learning methods are divided into two categories: unsupervised learning and supervised learning. In supervised learning, the model is trained using labeled input–output pairs [23]. In unsupervised learning, the model lacks a labeled dataset and only receives input data without known outputs. The model is tasked with independently deducing conclusions from the provided inputs [24]. The utilization of machine learning models in additive manufacturing processes is experiencing rapid growth. One valuable approach involves employing these models for in situ defect prediction to enhance quality control. This entails combining in-process optical tomography images with the initial part geometry and post-processing X-ray computer tomography data. Through this integration, the real-time formation of defects during the manufacturing process can be monitored, enabling the derivation of insights for optimizing processing parameters [25]. Splashes and delaminations were identified by Baumgartl et al. [26] via a convolutional neural network on thermographic image data. A further implementation of in situ thermographic data is shown by Estalaki et al. [27]. They presented a random forest classification model for the decision if a part is defect or normal on voxel state with high precision accuracy [27]. Furthermore, Du et al. [28] presented a physics-informed neural network to predict the balling effect of multiple materials. With their model, they revealed important variables for the occurrences of balling [28].

Considering the effects of defects on mechanical properties of additive manufactured components shows the urgency of deeper analysis of these defects. Often, there are no possibilities to use featured in situ machine learning models for acquiring defect information. Frequently, micrographs are necessary to examine microstructures, microhardness, or defect distributions. In this work, a model including the entire pipeline (image segmentation, feature extraction, and feature reduction) for the classifications of defects in laser additive manufactured parts based on micrographs is developed. This involves an investigation of unsupervised methods and their comparisons with supervised models. Binary images of segmented defects as well as locally extracted features of defects serve as input data. This model can be applied on micrographs even if computer tomography is not accessible. The information about the number of defects within the classes can also be used to investigate their behavior in thermal or thermal–mechanical post processing, as well as their influences on mechanical properties.

## 2. Materials and Methods

### 2.1. Data Generation and Feature Extraction

**Figure A1:**
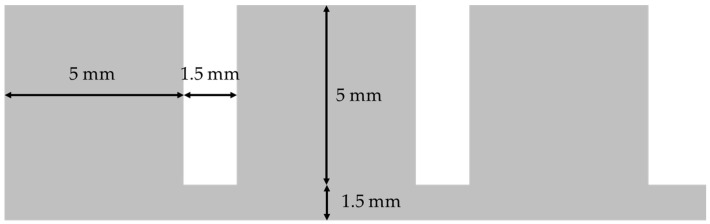
Specimen configuration and dimensions used for the data creation.

In order to apply unsupervised as well as supervised models to the problem of defect classification, a corresponding data pipeline and database had to be created and generated. For this purpose, a data set of manufacturing process parameter combinations for Ti6Al4V was used. This contains different laser powers, layer thicknesses, hatch distances, as well as scan speeds that are randomly distributed within given limits, Table 1. For efficient metallographic preparation, the specimens were manufactured in a configuration of three, connected by a small base. The size of each specimen segment is 5 × 5 × 6.5 mm^3^ (w × d × h), and the height of the base is 1.5 mm, Figure A1 in Appendix A. A broad interval of process parameters were deliberately selected in order to produce samples with a wide range of densities, defect type, sizes, and counts. In general, the layer thickness was fixed into four steps: 0.025 mm, 0.05 mm, 0.075 mm, and 0.1 mm. The other three process parameters were then randomly calculated following an equal distribution each and combined random too. Then, the energy density was used to prune the possible combinations between 6 J/mm^3^ and 200 J/mm^3^. This was applied to avoid extremely low and high energy inputs.

The data set was produced with CL 41 Ti ELI powder (Concept Laser GmbH, Lichtenfels, Germany) on an SLM 125 HL (SLM Solutions Group, Lübeck, Germany) and comprised a total of 816 parameter combinations, of which 770 could be built with one micrograph each. Due to the broad parameter study, a large number of different density values, between 31% and 100%, as well as defective morphologies and sizes, are available. The data set was produced in particular as a basis for machine learning methods. Such a large database with a high variance in the process parameters is not usual for a parameter study. Prior to the study, samples with contiguous (open) porosities were sorted out. From the remaining 635 micrographs, the defects were examined if any were present [29].

**Figure 1:**
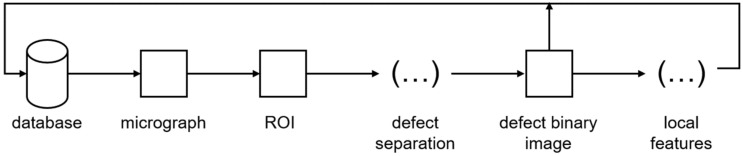
Pipeline for the extraction of the pore features and their binary images from the micrographs.

**Figure 2:**
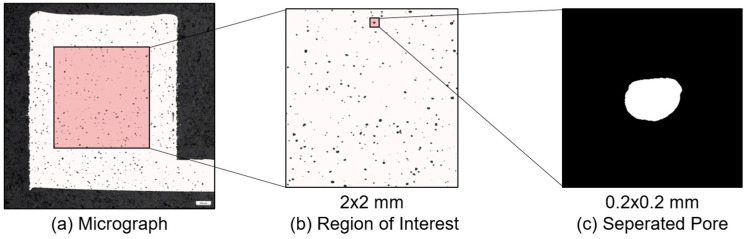
Extraction of single pores as binary images for model building and feature derivation.

The first step in the pipeline for data generation is to load a micrograph from the data set and to determine the region of interest (ROI) automatically by center cropping, Figure 1. Then, using a Python code with the OpenCV library [30], the images were binarized with the OTSU method, and, furthermore, the contours of the defects were determined. Each contour found in the region of interest (2 mm × 2 mm) was plotted and saved in an empty image of 360 PX × 360 PX (corresponding to 0.2 mm × 0.2 mm), Figure 2.

**Figure 3:**
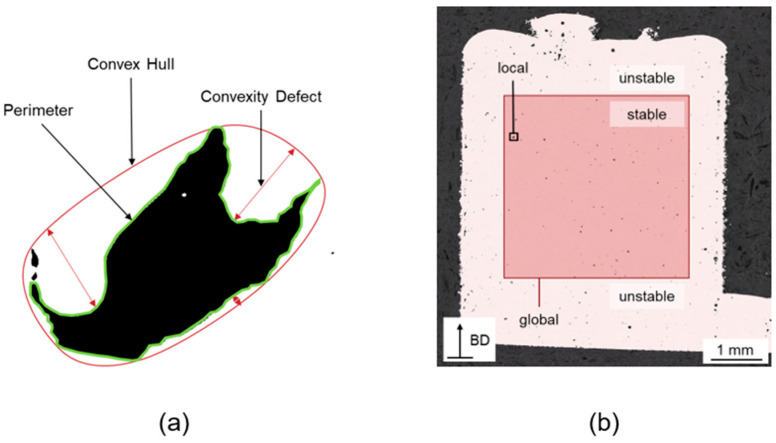
(**a**) Locally extracted features of the individual pores, and (**b**) representation of the ROI in the micrographs with the distinction between local and global features.

For each of the contours found the local features were determined, Figure 3a. In this work, local features were defect-related features. Global features are all features encompassing the entire region of interest, such as the relative density, the manufacturing process parameters, or the number of defects in relation to the measurement area, Figure 3b. The local features were generated and extracted directly by the used framework OpenCV. In addition, the following features can be calculated from these:

(2) $$Solidity=\frac{{Area}_{Contour}}{Area_{Conv.Hull}},$$

(3) $$Conv.Defect\;Density=\frac{{No}_{Conv.Defects}}{Area_{Contour}}.$$

The solidity calculated according to Equation (2) is the ratio of the real defect area enclosed by the perimeter to its convex hull, Figure 3a [31]. The closer this ratio is to the value of one, the higher the solidity or convexity of the defect. In addition, the density of convexity defects, according to Equation (3), was defined as the ratio of the number of defects to the defect area. Keyhole defects are assumed to be more convex in shape overall, although not circular. This convexity is described by the solidity. Furthermore, the density of the convexity defects as well as the mean size of the convexity defects are assumed to be reasonable features. Both describe the deviation of the defect shape from its convex hull.

**Figure 4:**
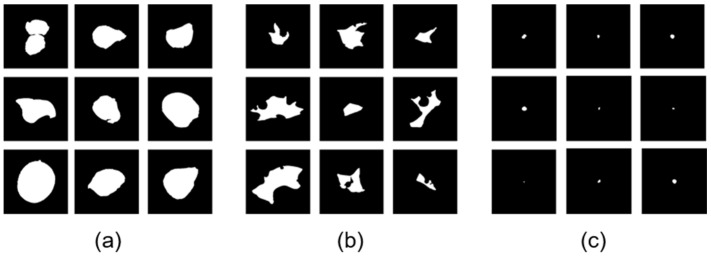
Extract from labeled dataset with defect binary images of (**a**) keyhole defects, (**b**) lacks of fusion defects, and (**c**) process pores.

In order to investigate steady process condition without influences of the building plate or the last layers in the building process, as well as edge effects on the contour of the specimens, the region of interest was set in the core of the specimen, Figure 3b. In this case, the square spanned from the center of the specimen. The complete feature extraction process was replicated for every micrograph within the dataset. In total, 90,066 defects were detected, segmented, and had their features extracted from the 635 micrographs. Experienced staff manually labeled some of the separated defects randomly chosen from different micrographs and stored as binary images. All the extracted defects were stored in a single folder, allowing staff to make selections. Consequently, there is no detailed information available regarding the specific number of micrographs or process parameters they may choose from. Distinctions were made in the categories of keyhole defects, lack of fusion defects, and process pores, Figure 4.

Further defect types could not be identified in the micrographs of the present data set. This results in a data set with 1200 instances: 400 per class with binary images, defect types, as well as the local features. This is especially needed for the training of the supervised models as well as for testing and comparing the models’ accuracies. Unsupervised models do not need this manual labeling procedure in general, but it will give more details about model decisions and generated classes applying them on new, unseen test data.

### 2.2. Modeling and Testing

Two types of models were employed for defect classification. Initially, unsupervised clustering algorithms (kMeans and DBSCAN) were utilized to determine if the models could independently differentiate between defect types without additional guidance. From a scientific perspective, this task is not a significant challenge, given the requisite expertise. Unsupervised methods, in this case, involve the use of kMeans and DBSCAN algorithms [32,33]. The task of both algorithms is to form clusters by assigning a set of data instances to groups. Similar points are assigned to a cluster, and the similarity between the clusters is minimized [21,22]. This clustering can be performed in different ways. The chosen models are among the most common models for unsupervised clustering of data. The kMeans algorithm is among the top 10 data mining algorithms [34]. This unsupervised clustering algorithm partitions a given dataset without any knowledge about it. In most cases, the number of distinguishable classes is unknown. By using validity indices, a suitable number of classes can be determined [35,36]. Due to its widespread popularity and user-friendly nature, this algorithm is becoming increasingly prevalent in various fields [25]. As physical processes are always subject to a certain amount of noise, the second chosen model is the DBSCAN algorithm. This algorithm is a density-based, unsupervised clustering algorithm specifically designed to group data of arbitrary shapes, even in the presence of noise [37]. During this process, data points are subdivided into core points, edge points, and outliers. This subdivision is based on the neighborhood radius (Epsilon) and the minimum number of neighbors (MinPts). If a point has the minimum number of neighbors in the radius Epsilon, it is classified as a core point. If a point has less than the minimum number of neighbors, but at least one core point in its neighborhood, it is an edge point. All other points are classified as outliers. All points adjacent to the core point are assigned to the same cluster [38]. Outliers found by the model can be, e.g., unfavorably cut pores. For this purpose, both the principal components of the pixel values and the extracted local features of the defects were used. The models were generated using the python framework Scikit-Learn [39]. This was followed by the training of a supervised model. For this purpose, a random forest tree classifier from the Scikit-Learn framework was selected [39]. The random forest tree model is a supervised learning algorithm. It involves generating a larger number of classifiers and combining their results. In this way, better predictions can be achieved than with a single classifier [40,41]. Random forest trees represent a well-known approach to pattern recognition [42]. In this approach, many decision trees are generated, which subsequently make the prediction by majority vote. In this process, random forest trees are shown to be robust and have a low tendency to overfitting. The number of decision trees is determined iteratively by trial and error. At a certain number of classifiers, the computational cost increases, but the model accuracy does not increase anymore [40]. The random forest tree classifier is also trained with both feature variants. The prediction accuracy was checked for a training dataset (70% of the instances) and a test dataset (30% of the instances).

**Figure 5:**
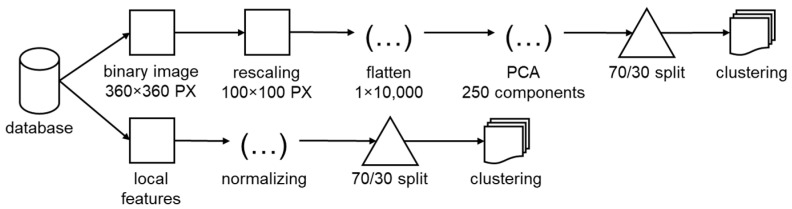
Pipeline for supervised defect clustering based on binary images and local features.

The data pipeline for the supervised training followed two strands. One for the binary images and one for the local features, Figure 5. The created database contained binary image data of 1200 defects and their corresponding local features. The images were resized to 100 PX by 100 PX and flattened in a 1 by 10,000 vector. 

Afterwards a principal component analysis was applied to reduce the 10,000 features to 250. These 250 principal components described the variance of the 10,000 features by 96.8%. The local features were simply normalized. Lastly, both strands included data splitting into training and test data. Finally, defects were extracted from unseen micrographs of Ti6Al4V and classified using the best trained model. Thus, more information on process parameter dependencies and the effects of defects on mechanical properties can be tapped.

## 3. Results and Discussion

### 3.1. Unsupervised Defect Classification with Cluster Algorithms on Binary Images

**Figure 6:**
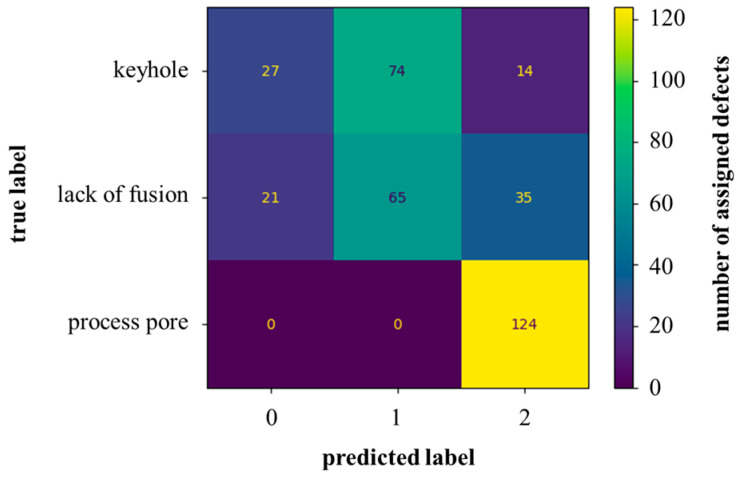
Confusion matrix of the unsupervised kMeans clustering model with 360 test data points corresponding to true labels; label 0–2 are generated by the model and do not correspond to the true labels.

For the unsupervised classification of the defects, the kMeans algorithm was employed in the first step. The 250 principal components were passed to the model as input data. Furthermore, the number of classes to be distinguished was set to three according to the labeled data set. The predicted labels were compared with the true labels in the confusion matrix, Figure 6. 

**Figure 7:**
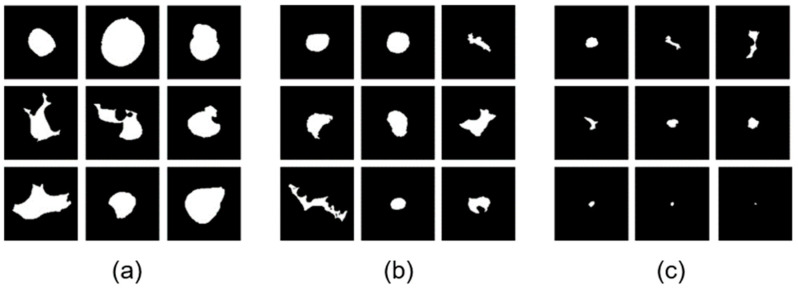
Results of unsupervised classification of defect binary images using the kMeans algorithm for a given number of three classes: (**a**) class 0, (**b**) class 1, and (**c**) class 2.

The model assigned most of the defects to class 2. All 124 process pores were assigned to this class. In total, 14 keyholes and 35 lacks of fusion defects were assigned to this class. The much smaller gas-induced round process pores could be relatively easily separated by the model. In the first class (Label 0), the model assigned 27 keyholes and 21 lacks of fusion defects. In the second class (Label 1), lack of fusion (65) and keyhole (74) defects were assigned, Figure 6. In summary, the kMeans algorithm sorted the defects into the three classes according to their sizes. The shapes of the defects seemed to determine the classification only to a small extent, Figure 7a–c.

**Figure 8:**
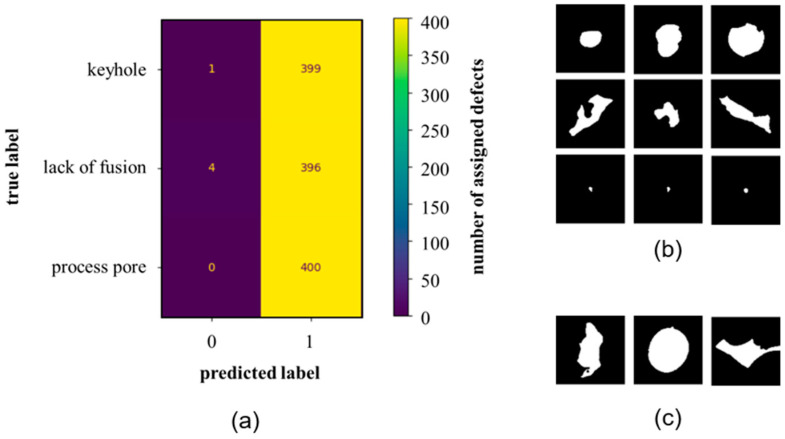
Results of unsupervised classification of defect binary images using DBSCAN algorithm: (**a**) Confusion Matrix, (**b**) DBSCAN Class 1, and (**c**) DBSCAN Class 0 (outliers).

The second unsupervised model is the DBSCAN algorithm. The 1200 data points in the forms of the 250 principal components were also given as features. The data were normalized to a value range [0, 1]. In contrast to kMeans, the DBSCAN model autonomously identified the number of clusters during the clustering process. The result showed that only one cluster was found. Almost all of the three defect types were assigned to class 1, Figure 8a. 

Only four lacks of fusion defects and one keyhole defect were assigned to class 0. This class included the outliers identified by the model. A discrimination scheme could not be discerned from the results. Despite variations in sizes and shapes, the data did not exhibit enough distinction to be effectively categorized by the model. The use of the pixel values (their principal components, respectively) covered all possible features for the descriptions of the defects. Despite this, the DBSCAN model did not reveal a significant distinction between the defect types. Additionally, the substantial number of features (250) may potentially hinder the results. However, it is noteworthy that the DBSCAN algorithm yielded similar outcomes when applied to the local features (5), indicating that dimensionality was not the primary challenge.

### 3.2. Supervised Defect Classification with Random Forest Trees on Binary Images

**Figure 9:**
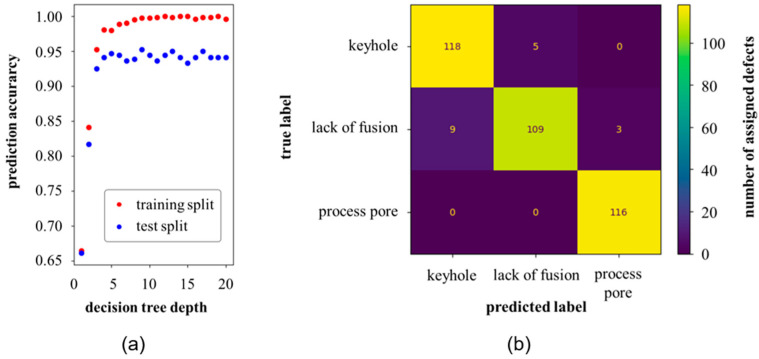
Random forest tree model trained on binary image data. (**a**) Accuracy at 15 estimators and a maximum of 100 features for the training and test data split over the decision tree depth and (**b**) confusion matrix from the best random forest tree classification model for the test data split at decision tree depth of 8 and a test accuracy of ~95%.

Through an iterative procedure, a total of 15 estimators (decision trees) were selected, each contributing to the classification of every defect. The depth of the estimators, i.e., the number of levels in the decision trees, was successively increased. Subsequently, the model with the highest prediction accuracy, with respect to both training and test data, was selected, which, at the same time, had a comparatively low depth, Figure 9a. The data set of 1200 instances was split into 70% training data and 30% test data. The training data set was used to generate/train the random forest tree models. Subsequently, the final model was tested with the reserved test data to evaluate its performance on previously unseen defects. From the matrix in Figure 9b, it can be seen that the model was capable of assigning the defect images to the correct labels with a very high accuracy of over 95% (for the unseen test data split). Only a few misclassified defects were detected in all classes.

### 3.3. Defect Classification with Random Forest Trees on Local Features

**Figure 10:**
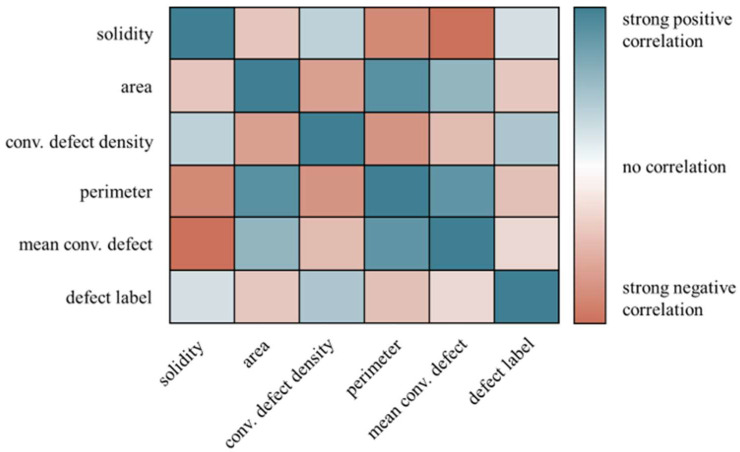
Correlation matrix of the local defect features and the manual labeled labels with coloring by the strength and sign of correlation, including the values of all labeled defects.

Employing pixel values as features provides a quick and straightforward approach. Conversely, opting for local features offers advantages such as reduced computation time and simplified data preparation. In this work, instead of dealing with 10,000 pixels, only five shape and size descriptive features were utilized. Before they were passed to the random forest tree model, the data were normalized to the range of values between 0 and 1. The correlation matrix showed that there were significant correlations between the local features of the defects and also between the local features and the defect labels, Figure 10. A high solidity was accompanied with a smaller defect area, perimeter, and mean convexity defect, ordered by increasing effect strengths. Further high solidities are connected to a higher convexity defect density. The areas of the defects increased with the mean convexity defect and perimeter and vice versa with decreasing solidity and convexity defect density. It could be observed that small defects exhibited higher solidity along with a denser population of high-convexity defects, yet they tended to have fewer convexity defects overall. This observation was particularly relevant when describing process pores, which typically manifested as small, spherical-shaped defects. The increase in convexity defect densities was a natural consequence of a reduced area, as indicated by Equation (3) (see Figure 10). Perhaps considering solely the count of convexity defects could serve as a more informative feature for pore classification. Defects exhibiting a high mean convexity value, along with larger area and perimeter, alongside low solidity and convexity defect density, were indicative of lacks of fusion defects. These defects were characterized by substantial areas and prominent convexity defects. Considering the sizes of these defects, the low convexity defect densities could be explained. Gaining insights into keyhole defects was not straightforward, as their sizes and shapes typically fell between those of process pores and lacks of fusion defects. Any visible correlations between the defect labels and the local features should be approached with caution, primarily because the labels represented classes rather than numerical values. For modeling purposes, these classes were assigned numerical values (0, 1, and 2), which could potentially introduce misleading correlations within the data matrix.

**Figure 11:**
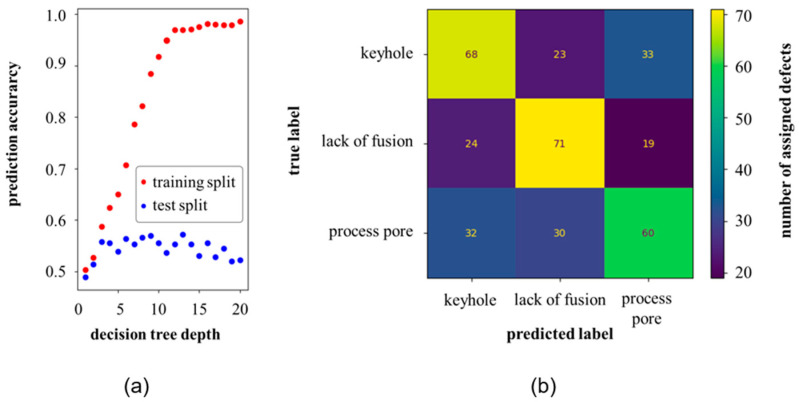
(**a**) Prediction accuracy for the training and test data over the decision tree depth by use of the local features, and (**b**) confusion matrix of the best trained random forest tree model for the test data split with a decision tree depth of 13 and a test data accuracy of ~55%.

For the random forest tree model based on the local features, an optimal number of 15 estimators resulted after an iterative procedure. With a depth of 13 levels, the model showed the highest accuracy of about 55%. Compared to the random forest tree model with the pixel values as inputs, the model trained on the local features showed significantly lower prediction accuracies for the test data split, Figure 11a.

The confusion matrix gave a more detailed account of the predictions of the model, Figure 11b. Compared to the model based on pixel values, the model based on local features showed inaccuracies for all three defect types. Out of the keyhole defects, about 55%, the lacks of fusion defects were about 62%, and the process pores, about 49%, were assigned to the correct labels. The model’s performance showed a pronounced increase in error when exclusively relying on local features. Furthermore, it was evident that there was a substantial gap between the prediction accuracy on the training data and that on the test data, with notably higher accuracy observed for the former. The model fit well on the training data but not on the test data. Excessive model complexity tended to be ruled out due to the hyperparameters chosen. Due to the strong feature reductions (from pixel values to five shape-describing local features), the model may require a larger set of instances (>1200) or more/better features for high generalization.

### 3.4. Comparison of the Models

All presented models, unsupervised and supervised, were trained with the training data and split and tested with the test data split. By this, they could be compared regarding their accuracies on the defect classifications of unseen data. In Table 2, the accuracies for the kMeans and the two random forest tree classifiers (on image data and on the local features) are presented. The overall prediction accuracy was the highest for the random forest tree classifier trained on defect image data with 95.28%. This was followed by the kMeans model with 60% and the random forest tree trained on local features with 55.28% accuracy. Evaluating the accuracies concerning defect types revealed significant differences between the models. The process pores could be classified with 100% accuracy by the kMeans and the image-based random forest tree classifier. The random forest tree classifier based on the local features offered only an accuracy of 49.18% for these defects. Except for the image-based random forest tree, both other models showed significantly lower accuracies for lack of fusion and keyhole defects. Also, the image-based random forest tree classifier showed lower accuracies for these types of defects with 90.08% and 95.93%, which were very high anyway.

Overall, the image-based random forest tree showed the best and good results in classifying defects resulting from the laser powder bed fusion process. Using manual, staff-labeled data for supervised training improved the decidability of the model between lacks of fusion and keyhole defects. These types of defects could have similar attributes sometimes. The smooth transition between those two types could be the reason for the failures of the kMeans model and the lower accuracies of the random forest tree classifier on those to defect types.

### 3.5. Model Application on Unlabeled PBF-LB/M Fabricated Ti6Al4V Micrographs

**Figure 12:**
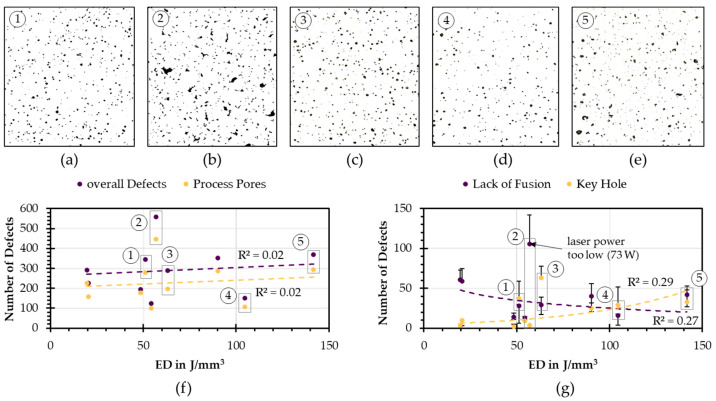
(**a**–**e**) Micrographs of five different process parameter combinations, (**f**) total number of defects and process pores over the energy density, positions of the micrographs (**a**–**e**) are marked with their number and (**g**) absolute number of classified lack of fusion and keyhole defects over the energy density with numbers of selected micrographs. Outlier with too low laser power, excluded for regression model, marked with the number 2.

In the following, the model was applied on 10 selected micrographs for process parameters with a constant layer thickness of 50 µm. The parameter combinations resulted in different volume energy densities, Table A1 in Appendix A. The defect classification pipeline was implemented on these micrographs, and, subsequently, the classification results were reviewed by the staff to assess the model’s accuracy. Incorrectly classified defects were represented by negative error bars, whereas positive error bars denoted missing defects that were classified into the wrong classes, Figure 12g. The overall classification accuracy on all 10 images was 92% is slightly lower than that for the test data split shown before.

The number of defects, in general, increased slightly with the energy density. The number of process pores increased parallel to the overall number of defects, Figure 12f. With increasing energy density, the number of lack of fusion defects decreased, whereas the number of keyhole defects increased, Figure 12g. Significant errors were observed for lack of fusion and keyhole defects, leading to low R2 values, Figure 12g. In Figure 12b, large lacks of fusion defects, resulting from very low laser power of 73 W, became evident. This suggested why this data point was such a strong outlier compared to the other lack of fusion defects, Figure 12g. Figure 12a is the result of medium energy density and a laser power scan speed ratio of ~0.5 J/mm paired with a high hatch distance, forming many keyhole defects. Here, the model also detected a large amount of lack of fusion defects, which indicated that these keyhole defects were more irregular shaped than typically shaped. This could be observed for multiple of the studied parameter combinations, parameter 3 Figure 12c, parameter 4 Figure 12d, and parameter 5 Figure 12e.

Another notable finding is that a high energy density, in this case 104 J/mm^3^, in combination with a low hatch distance of 0.07 mm, resulted in lower amounts of defects in general (see Figure 12f ④) with a higher number of unshaped keyhole defects. This leads to a higher rate of false classification as lack of fusion defects (see Figure 12g ④). Same can be observed for a very high energy density paired with a high ratio of laser power and scan speed (0.83 J/mm) and medium hatch distance. Although this led to a general increasing number of defects, it also resulted in the emergence of larger irregularly shaped keyhole defects, which were frequently misclassified as lacks of fusion defects by the model.

## 4. Conclusions

In this work, the automated classification of defects of laser additive manufactured components made of Ti6Al4V was investigated. For this purpose, a pipeline for the extraction of local features of defects, as well as the separation of defect binary images from micrographs, was developed.

- Unsupervised models like the kMeans and DBSCAN algorithm failed in classifying process pores, keyholes, and lacks of fusion defects. However, a supervised random forest tree classifier showed high accuracies, around 96%, for the classification. The staff-labeled data were needed, so the models could distinguish especially between the lacks of fusion and keyhole defects.
- The supervised classification models performed better on binary image data of defects than on locally extracted features describing their morphologies. More local features are needed to achieve better classification results.
- Depending on the process parameters used in additive manufacturing, keyhole defects could become more irregular and unshaped even at relatively low energy densities. This resulted in major classification errors between lacks of fusion and keyhole defects. Considering the influences of the defects on mechanical properties, even keyhole defects could possibly significantly reduce the mechanical properties if they were more irregular and unshaped.

## 5. Outlook

Employing the model alongside manual verification of predicted results enables dataset expansion and contributes to improving the model’s accuracy. In particular for the separation of keyhole and lacks of fusion defects. Furthermore, the model could be retrained for three-dimensional data, such as computer tomography images, which may also improve the model’s accuracy. Finally, the local features presented and additional ones could be used to describe the process parameters’ influences on keyhole defects, why or under which conditions they become more irregular and unshaped, and how these influence mechanical properties.

## Figures and Tables

**Table 1:** Process parameter range for Ti6Al4V parameter study with count as the number of unique values of each process parameter; constant parameters were: Spot size of 66 µm, build plate temperature of 200 °C, stripes scan pattern, and layer rotation of 67°.

	Laser Power *P_L_* in W	Layer Thickness *D_L_* in µm	Scan Speed *v_S_* in mm/s	Hatch Distance *d_H_* in mm
Min	51	0.025	201	0.05
Max	350	0.1	1700	0.2
Count	290	4	628	794

**Table 2:** Comparison of the results of the possibly usable models investigated in this work, containing one unsupervised and the two supervised models’ accuracies for the test data split in general and for each defect class separately (random forest tree abbreviated as RFT).

Model	Accuracy in %			
	Overall	Process Pores	Lack of Fusion	Key Hole
kMeans	60	100	57.75	22.5
RFT images	95.28	100	90.08	95.93
RFT local features	55.28	49.18	62.28	54.84

## Data Availability

We provide the code at DOI 10.5281/zenodo.8290175 and the data at DOI 10.5281/zenodo.8303011.

## Appendix A

**Table A1:** Process parameters, energy densities and relative densities of the validation dataset.

Label	$d_{H}$	$P_{L}$	$v_{S}$	$D_{L}$	ED	Rel. Density
-	mm	W	mm/s	mm	J/mm^3^	%
1	0.19	134	283	0.05	51.2	98.12
2	0.12	73	225	0.05	56.89	96.76
3	0.11	188	565	0.05	63.18	97.24
4	0.07	250	690	0.05	104.60	98.36
5	0.12	254	306	0.05	141.70	97.65
6	0.18	317	668	0.05	54.35	99.45
7	0.12	104	367	0.05	48.43	98.75
8	0.14	278	452	0.05	90.24	97.92
9	0.20	195	958	0.05	20.55	96.30
10	0.19	319	1693	0.05	19.65	97.45
